# Supplementary material for: KIF1C, an RNA transporting kinesin-3, undergoes liquid-liquid phase separation through its C-terminal disordered domain
Source: bioRxiv. 2023 Oct 23:2023.10.23.563538. Preprint. [Version 1] doi: 10.1101/2023.10.23.563538 (PMC10634753; doi:10.1101/2023.10.23.563538)
Supplement: 1 [file NIHPP2023.10.23.563538V1-supplement-1.pdf]

bioRxiv preprint doi: https://doi.org/10.1101/2023.10.23.563538; this version posted October 23, 2023. The copyright holder for this preprint (which was not certified by peer review) is the author/funder, who has granted bioRxiv a license to display the preprint in perpetuity. It is made available under aCC-BY 4.0 International license.

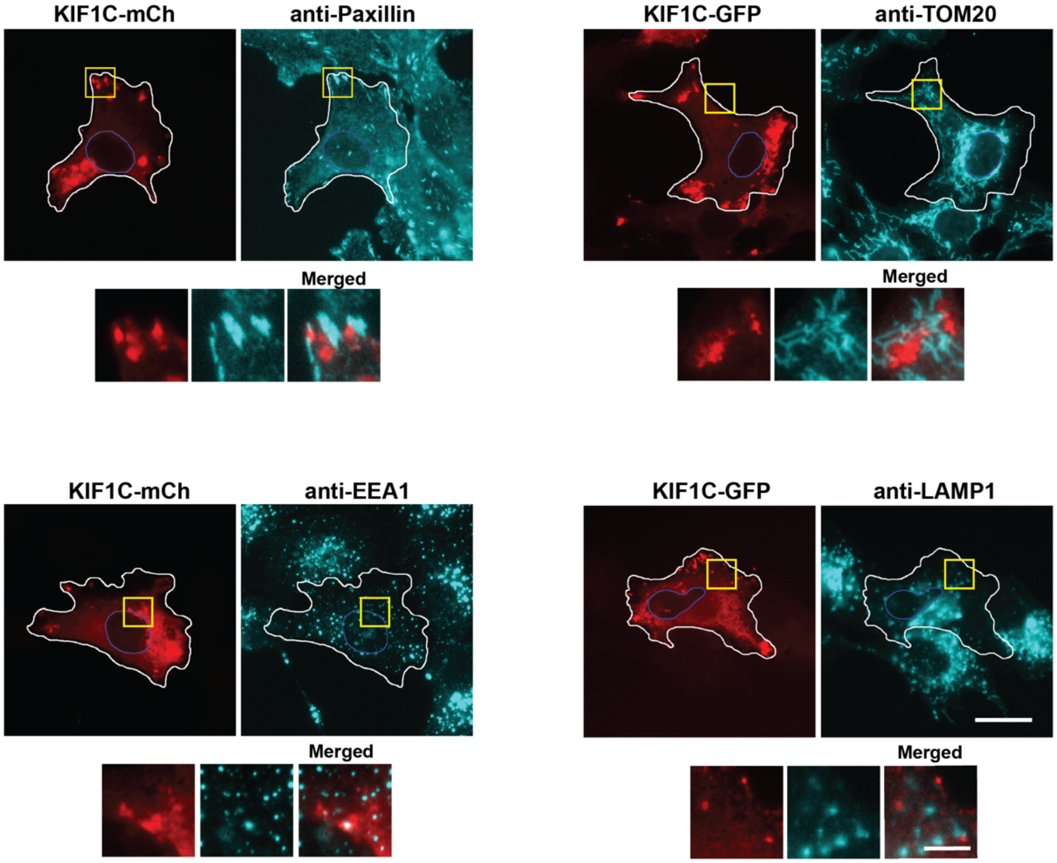

Figure S1. KIF1C puncta do not colocalize with organelle markers.
Immunofluorescence of markers for focal adhesions (Paxillin), early endosomes (EEA1), mitochondria (TOM20), and lysosomes (LAMP1) in hTERT-RPE1 cells expressing fluorescently-tagged KIF1C. Representative images are shown. White lines indicate cell boundaries. Blue lines indicate nuclear boundaries. Yellow boxes indicate regions shown in magnified images below. Scale bar: 20 µm for whole cell views, 5 µm for magnified images.

bioRxiv preprint doi: https://doi.org/10.1101/2023.10.23.563538; this version posted October 23, 2023. The copyright holder for this preprint (which was not certified by peer review) is the author/funder, who has granted bioRxiv a license to display the preprint in perpetuity. It is made available under aCC-BY 4.0 International license.

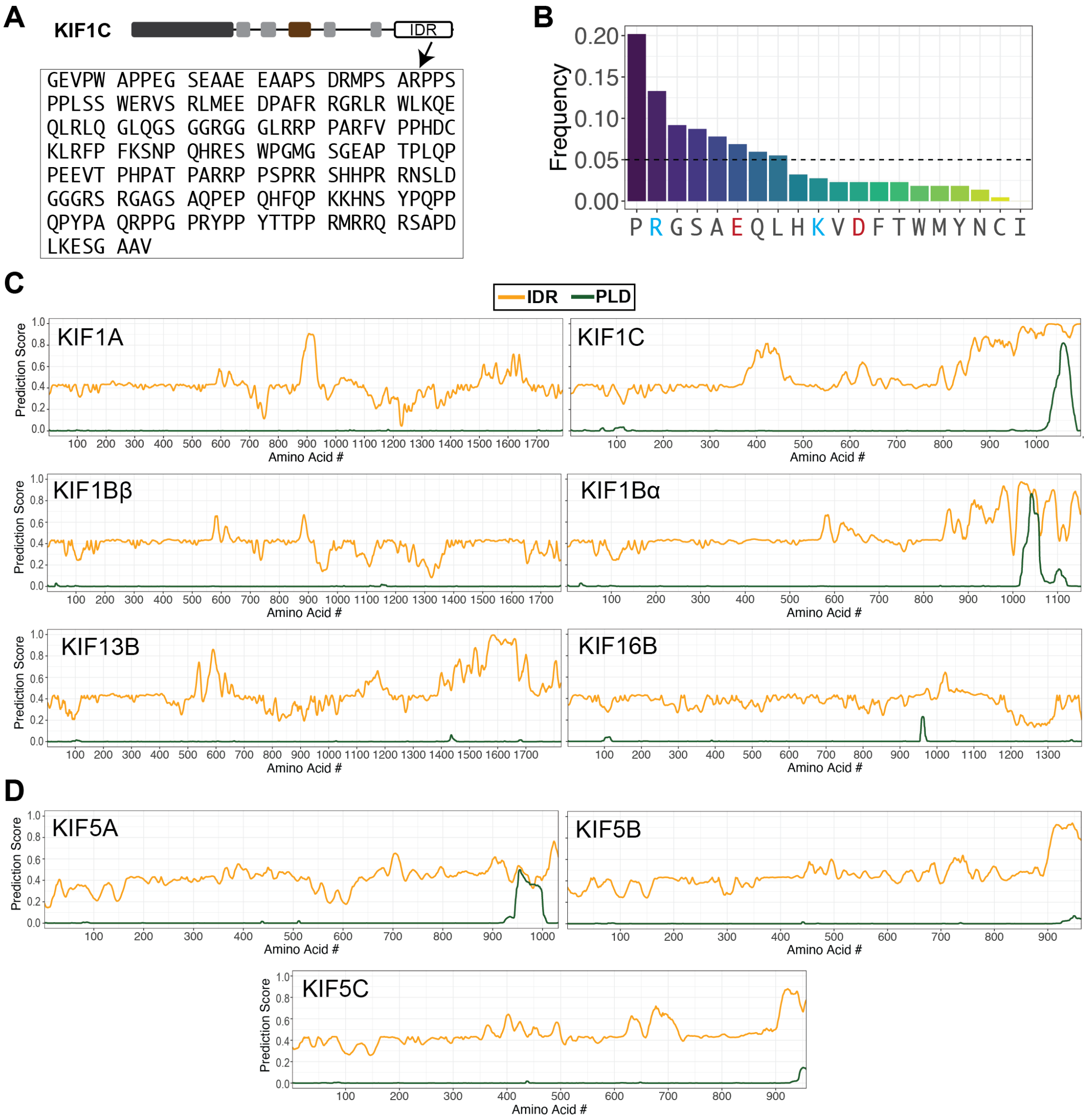

Figure S2. The KIF1C tail domain is an IDR.

(A) Amino acid sequence of the KIF1C IDR.

(B) Frequency of amino acid residues in the KIF1C IDR. The horizontal dashed line indicates the frequency of 0.05. For the x-axis, the positively charged residues R and K are labelled blue; the negatively charged residues E and D are labelled red.

(C,D) IUPred and PLAAC predictions of IDR and PLD, respectively, for (C) kinesin-3 family members and (D) kinesin-1 family members. x-axis: amino acid residue number; y-axis: predicted probability of the given residue being part of an IDR (orange line) or a PLD (green line).

bioRxiv preprint doi: https://doi.org/10.1101/2023.10.23.563538; this version posted October 23, 2023. The copyright holder for this preprint (which was not certified by peer review) is the author/funder, who has granted bioRxiv a license to display the preprint in perpetuity. It is made available under aCC-BY 4.0 International license.

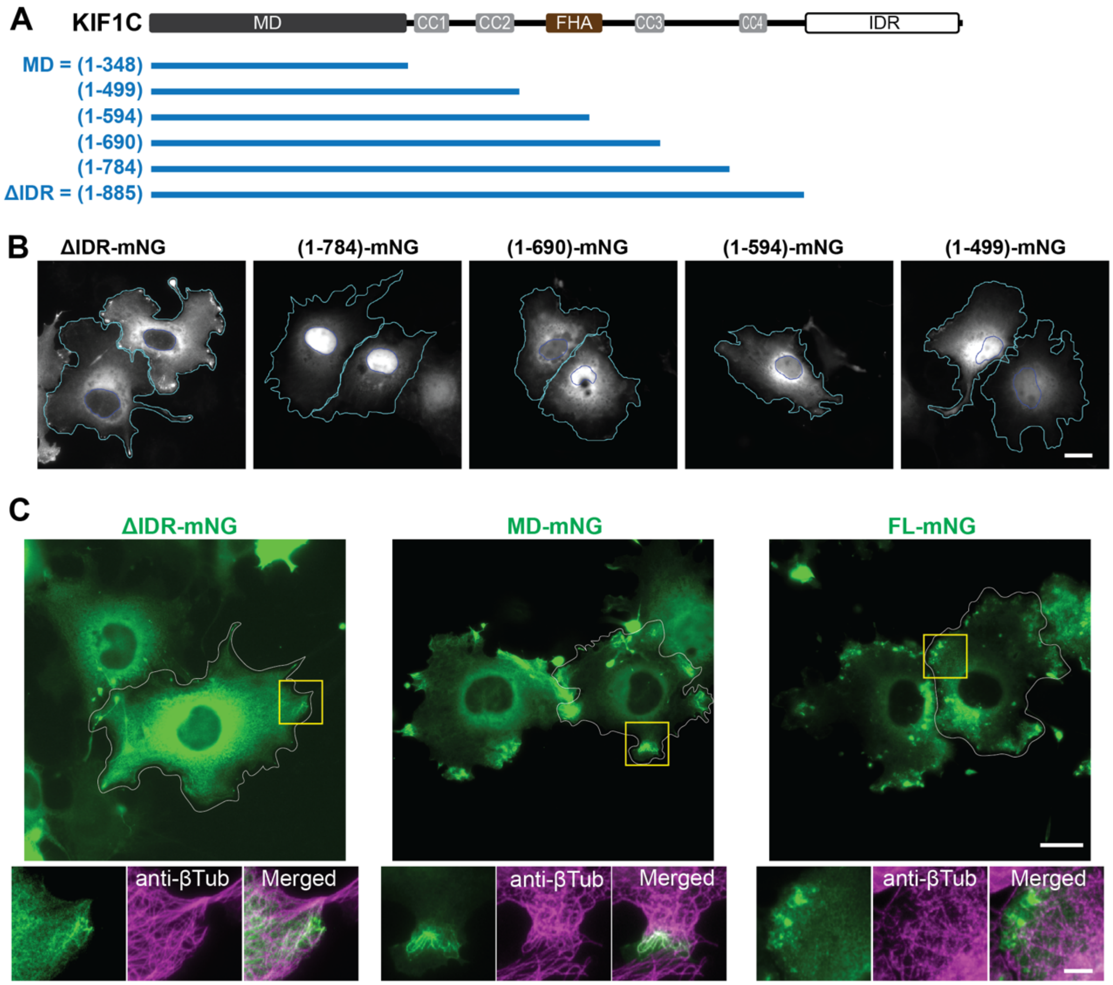

Figure S3. The IDR is required for KIF1C puncta formation in cells.
(A) Schematic of KIF1C serial truncations from the C-terminus.
(B) Representative images of KIF1C truncations in COS-7 cells. Scale bar: 20 μm. Cyan lines indicate cell boundaries. Blue lines indicate nuclear boundaries.
(C) Immunofluorescence of microtubules (anti-βTubulin) in COS-7 cells expressing KIF1C(ΔIDR)-mNG, KIF1C(MD)-mNG, or KIF1C(FL)-mNG. Scale bar: 20 μm for whole cell views, 5 μm for magnified images.

bioRxiv preprint doi: https://doi.org/10.1101/2023.10.23.563538; this version posted October 23, 2023. The copyright holder for this preprint (which was not certified by peer review) is the author/funder, who has granted bioRxiv a license to display the preprint in perpetuity. It is made available under aCC-BY 4.0 International license.

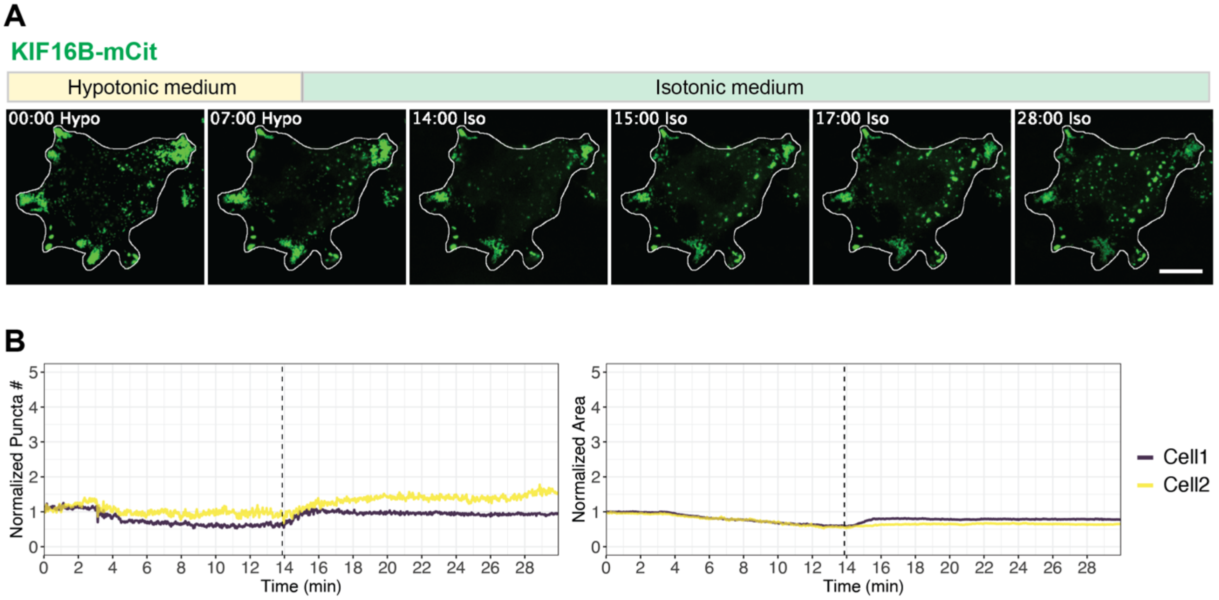

Figure S4. KIF16B does not change during the cytoplasm dilution assay.
(A) Representative images of mCit-tagged KIF16B localization before treatment, during hypotonic treatment, and upon return to isotonic media. Scale bar: 20 µm. Time label is [min:sec].
(B) Quantification of change of puncta number (left) and total area of puncta (right) over time in the cytoplasm dilution assay. x axis: time, with a vertical dashed line indicating the time point of switching from the hypotonic media to the isotonic media. y axis: puncta number or puncta area normalized against the initial state in the first frame of live-cell imaging. The example cell in (A) is Cell 1 in the plots.

bioRxiv preprint doi: https://doi.org/10.1101/2023.10.23.563538; this version posted October 23, 2023. The copyright holder for this preprint (which was not certified by peer review) is the author/funder, who has granted bioRxiv a license to display the preprint in perpetuity. It is made available under aCC-BY 4.0 International license.

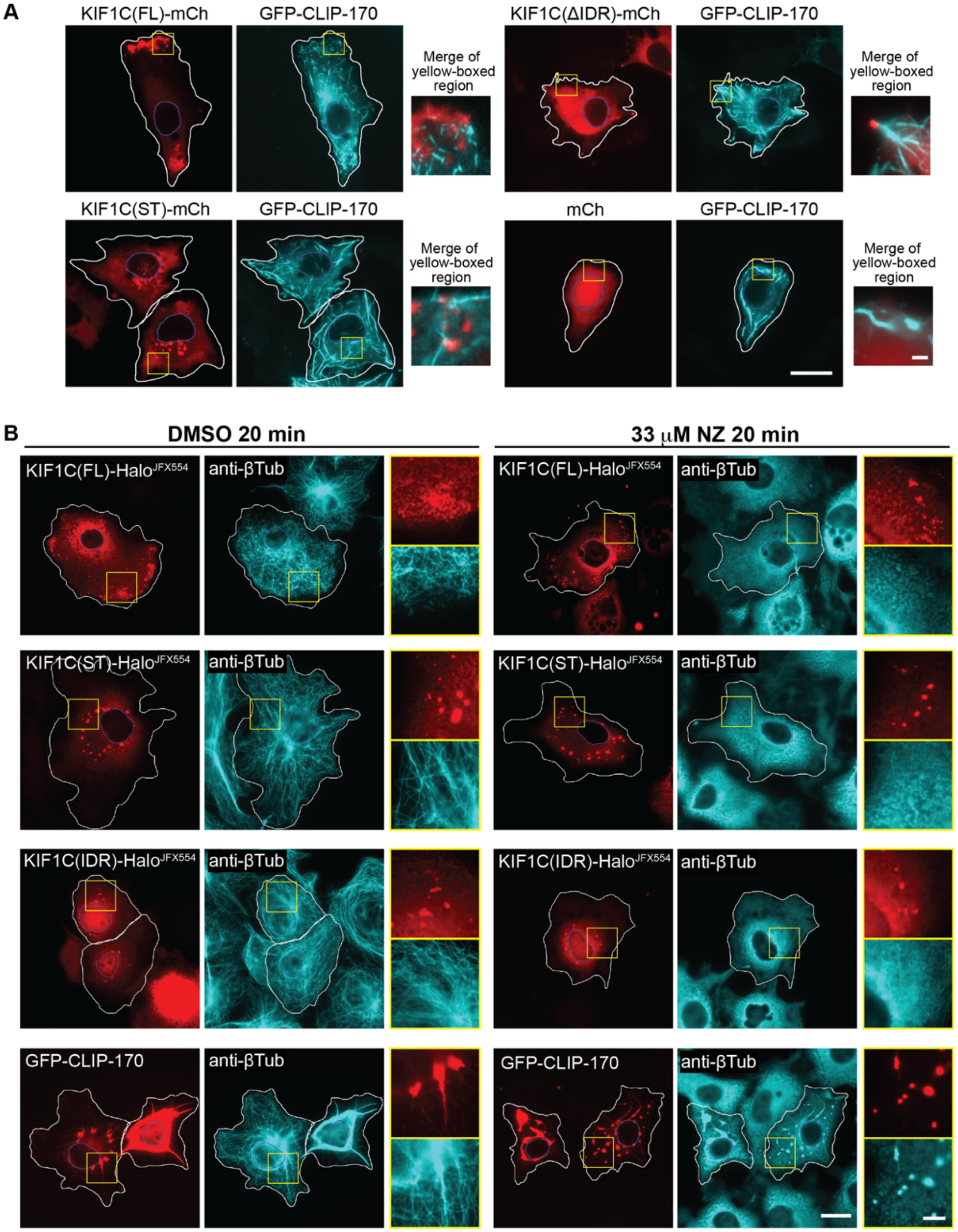

Figure S5. KIF1C condensates do not colocalize with CLIP-170 or tubulin.
(A) Localization of mCh (control) or mCh-tagged KIF1C constructs (FL, ST, and ΔIDR) co-expressed with GFP-tagged CLIP-170 in hTERT-RPE1 cells. Representative images are shown. White lines indicate cell boundaries. Blue lines indicate nuclei boundaries. Yellow boxes indicate the regions displayed in the magnified images to the right. Scale bars: 20 µm for whole cell images, 2 µm for magnified images.
(B) Immunofluorescence of anti-βTubulin in COS-7 cells expressing $Halo^{JFX554}$-tagged KIF1C constructs (FL, ST, and IDR) or GFP-tagged CLIP-170. Cells were treated with (left) DMSO or (right) 33 µM nocodazole (NZ) for 20 min. White lines indicate cell boundaries. Yellow boxes indicate the regions displayed in the magnified images to the right. Scale bars: 20 µm for whole cell images, 5 µm for magnified images.

bioRxiv preprint doi: https://doi.org/10.1101/2023.10.23.563538; this version posted October 23, 2023. The copyright holder for this preprint (which was not certified by peer review) is the author/funder, who has granted bioRxiv a license to display the preprint in perpetuity. It is made available under aCC-BY 4.0 International license.

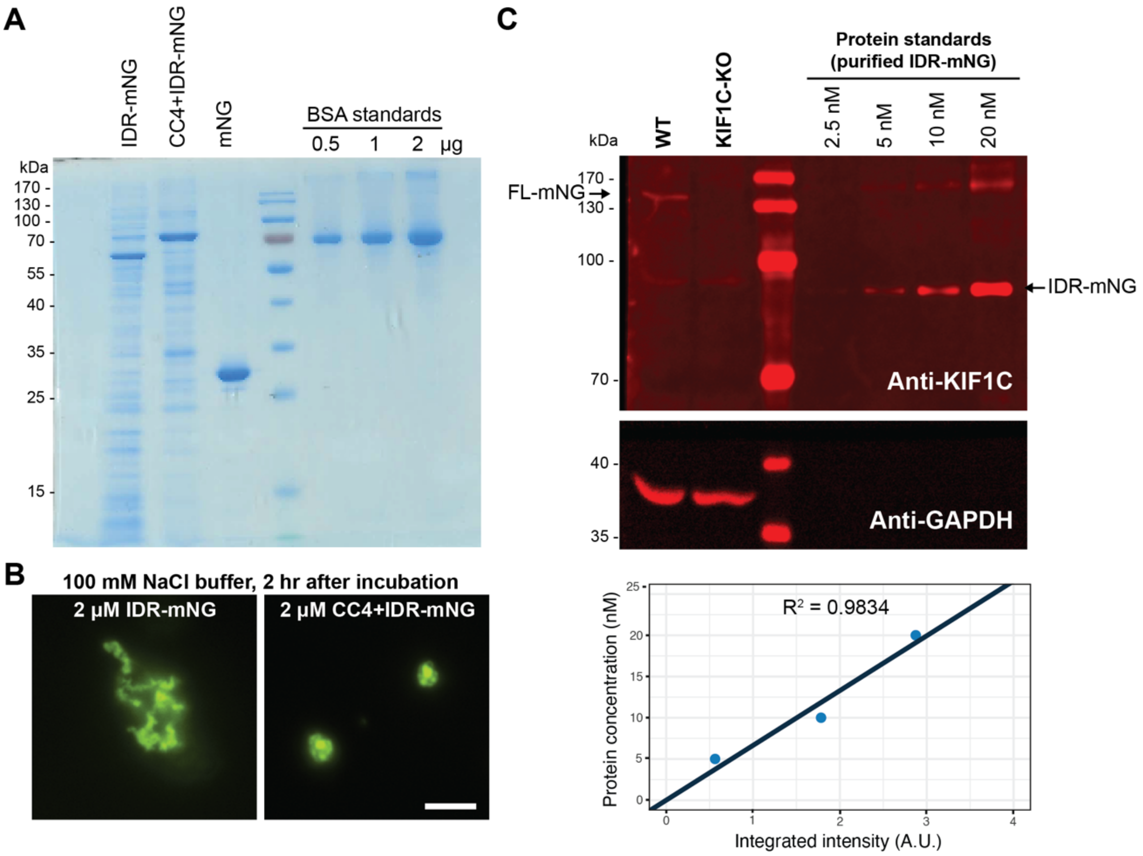

Figure S6. LLPS of purified IDR and CC4+IDR proteins.

(A) Purified KIF1C(IDR)-mNG, KIF1C(CC4+IDR)-mNG, and mNG separated by SDS-Page. Bovine serum albumin (BSA) was run as protein standards.

(B) Representative images of purified KIF1C(IDR)-mNG and purified KIF1C(CC4+IDR)-mNG imaged after prolonged incubation (2 hours). Scale bar: 5 μm.

(C) Estimation of endogenous KIF1C concentration in hTERT-RPE cells by western blot. Cell lysates of WT and KIF1C-KO hTERT-RPE1 cells were probed by western blotting with an antibody against KIF1C and against GAPDH (loading control). Purified KIF1C(IDR)-mNG was used as protein standards to generate a standard curve (bottom) for calculating the concentration of endogenous KIF1C.

bioRxiv preprint doi: https://doi.org/10.1101/2023.10.23.563538; this version posted October 23, 2023. The copyright holder for this preprint (which was not certified by peer review) is the author/funder, who has granted bioRxiv a license to display the preprint in perpetuity. It is made available under aCC-BY 4.0 International license.

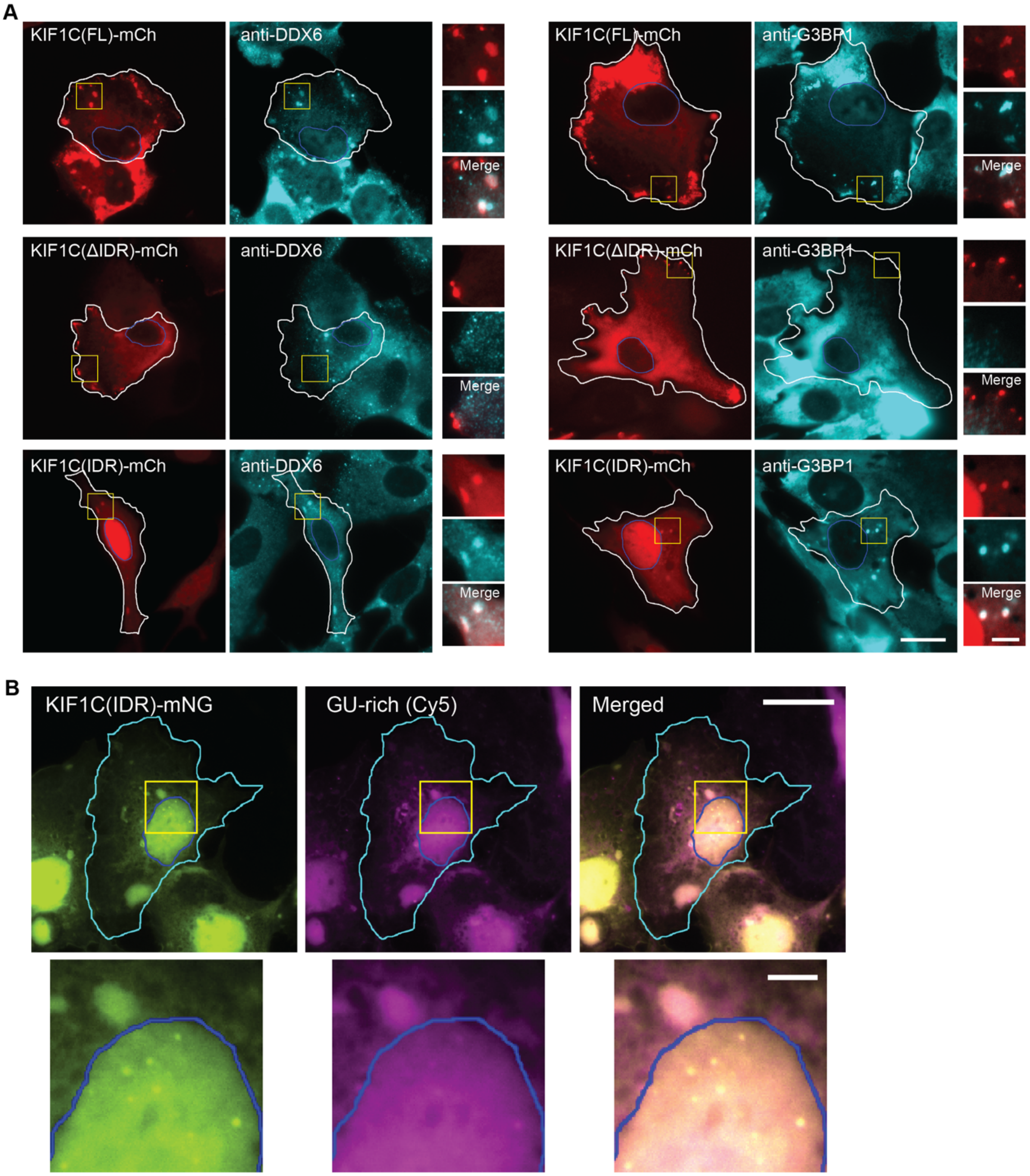

Figure S7. KIF1C colocalizes with endogenous RNA granules and exogenous RNA.
(A) Immunofluorescence for markers for P-bodies (anti-DDX6) and stress granules (anti-G3BP1) in hTERT-RPE1 cells expressing mCh-tagged KIF1C(FL), KIF1C(ΔIDR), or KIF1C(IDR). Representative images are shown. White lines indicate cell boundaries. Blue lines indicate nuclear boundaries. Yellow boxes indicate the regions displayed in the magnified images to the right. Scale bar: 20 μm for whole cell views, 5 μm for magnified images.
(B) Representative images of Cy5-labelled GU-rich RNA oligos introduced into COS-7 cells expressing KIF1C(IDR)-mNG. Yellow boxes indicate the regions displayed in the magnified images on the bottom. Cyan lines indicate cell boundaries. Blue lines indicate nuclear boundaries. Scale bars: 20 μm for whole cell views, 5 μm for magnified images.

bioRxiv preprint doi: https://doi.org/10.1101/2023.10.23.563538; this version posted October 23, 2023. The copyright holder for this preprint (which was not certified by peer review) is the author/funder, who has granted bioRxiv a license to display the preprint in perpetuity. It is made available under aCC-BY 4.0 International license.

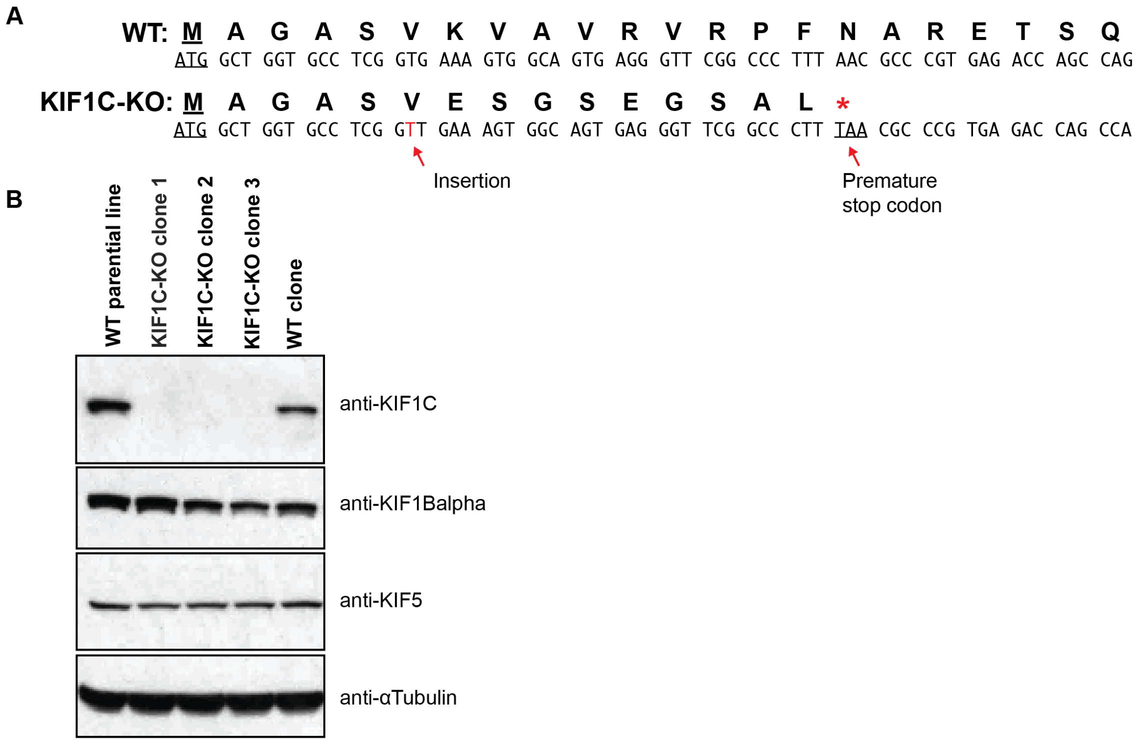

Figure S8. Verification of KIF1C-KO in hTERT-RPE1 cells.
(A) DNA and protein sequences from the start codon (underlined) in WT (top) and KIF1C-KO (bottom) hTERT-RPE1 cells. The same insertion of T (red text) was found in all 3 clones of KIF1C-KO cells, which results a premature stop codon (underlined and with a red asterisk).
(B) Western blot showing the absence of KIF1C protein in all three KIF1C-KO clones.

bioRxiv preprint doi: https://doi.org/10.1101/2023.10.23.563538; this version posted October 23, 2023. The copyright holder for this preprint (which was not certified by peer review) is the author/funder, who has granted bioRxiv a license to display the preprint in perpetuity. It is made available under aCC-BY 4.0 International license.

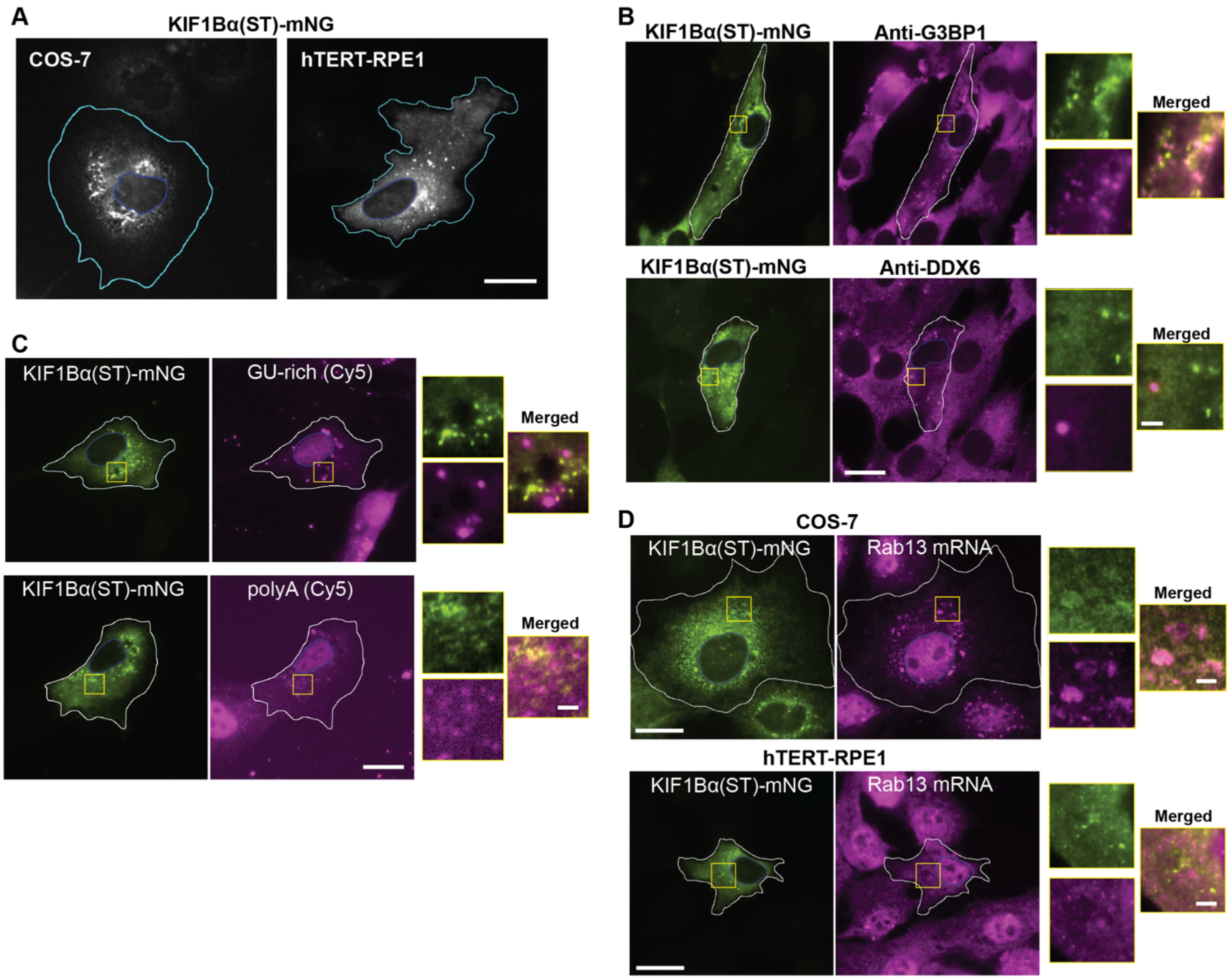

Figure S9. Characterization of KIF1Bα(ST) puncta.

(A) Localization of KIF1Bα(ST)-mNG in COS-7 cells and hTERT-RPE1 cells. Representative images are shown. Cyan lines indicate cell boundaries. Scale bar: 20 µm.

(B) Immunofluorescence for markers for stress granules (anti-G3BP1) or P-bodies (anti-DDX6) in hTERT-RPE1 cells expressing KIF1Bα(ST)-mNG. Representative images are shown. White lines indicate cell boundaries. Yellow boxes indicate the regions displayed in the magnified images to the right. Scale bar: 20 µm for whole cell views, 5 µm for magnified images.

(C) Representative images of Cy-5-labelled GU-rich or polyA RNA oligos introduced into hTERT-RPE1 cells expressing KIF1Bα(ST)-mNG. White lines indicate cell boundaries. Yellow boxes indicate the regions displayed in the magnified images to the right. Scale bars: 20 µm for whole cell views, 2 µm for magnified images.

(D) Representative images of KIF1Bα(ST)-mNG localization in COS-7 cells (top) or hTERT-RPE1 cells (bottom) with smFISH for endogenous *Rab13* mRNA. White lines indicate cell boundaries. Blue lines indicate nuclear boundaries. Yellow boxes indicate the regions displayed in the magnified images to the right. Scale bars: 20 µm for whole cell views, 2 µm for magnified images.

bioRxiv preprint doi: https://doi.org/10.1101/2023.10.23.563538; this version posted October 23, 2023. The copyright holder for this preprint (which was not certified by peer review) is the author/funder, who has granted bioRxiv a license to display the preprint in perpetuity. It is made available under aCC-BY 4.0 International license.

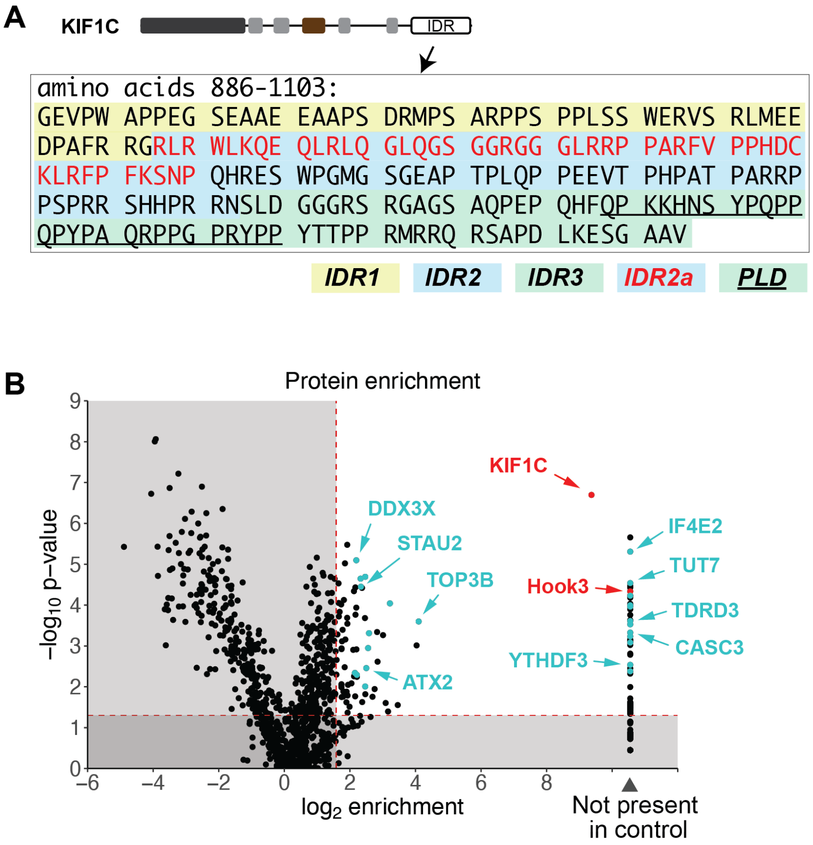

Figure S10. KIF1C-BioID identified numerous RBDs as interaction partners.

(A) Amino acid sequence of the KIF1C IDR with IDR1, IDR2, and IDR3 subregions color-coded. Subregion IDR2a within IDR2 is highlighted by red text. Subregion PLD within IDR3 is underlined.

(B) Volcano plot showing proteins identified in KIF1C-BioID experiments. Image modified from Kendrick et al., 2019, Figure 1D (reference 57). Proteins with an enrichment ratio > 3 and a p-value < 0.01 are included in the list. The interaction between KIF1C and Hook3 proteins (red text) has been characterized by Siddiqui et al., 2019 and Kendrick et al., 2019 (references 56 and 57). The cyan spots indicated RBPs identified in the KIF1C interactome. The cyan text indicates RBPs involved in mRNA processing or decay.
